# Climate Change Adaptation: Weighing Strategies for Heat-Related Health Challenges

**DOI:** 10.1289/ehp.121-a134

**Published:** 2013-04-01

**Authors:** Julia R. Barrett

**Affiliations:** Julia R. Barrett, MS, ELS, a Madison, WI–based science writer and editor, has written for *EHP* since 1996. She is a member of the National Association of Science Writers and the Board of Editors in the Life Sciences.

Many public health impacts have been predicted for climate change, but there has been relatively little exploration of ways to minimize the risks and develop long-term adaptation strategies. A new overview outlines the critical elements needed to address one such impact: heat-related illnesses and deaths, which are expected to increase with more frequent and more intense heat waves [*EHP* 121(4):415–419; http://dx.doi.org/1206025].

Adapting to the anticipated weather extremes arising from climate change has two main thrusts: reducing exposure and managing health risks. Reducing heat exposure often means having access to air conditioning, but widespread use of air conditioning can overburden the electrical grid to the point of large-scale outages. It can also further pollute the atmosphere as power plants increase output to meet electricity demands.

Reliance on air conditioning could be reduced through better building design and use of materials that reflect heat or insulate against it in new construction and renovations. These methods could be incorporated in urban planning that also encompasses green spaces (i.e., trees and open areas) and other measures known to mitigate the tendency for urban areas to trap and retain heat. Health risks might be mitigated to some degree through real-time health data surveillance, which would enable officials to detect crises early and warn the public accordingly. But health care facilities will need to be prepared for increased demand during heat waves.

**Figure:**
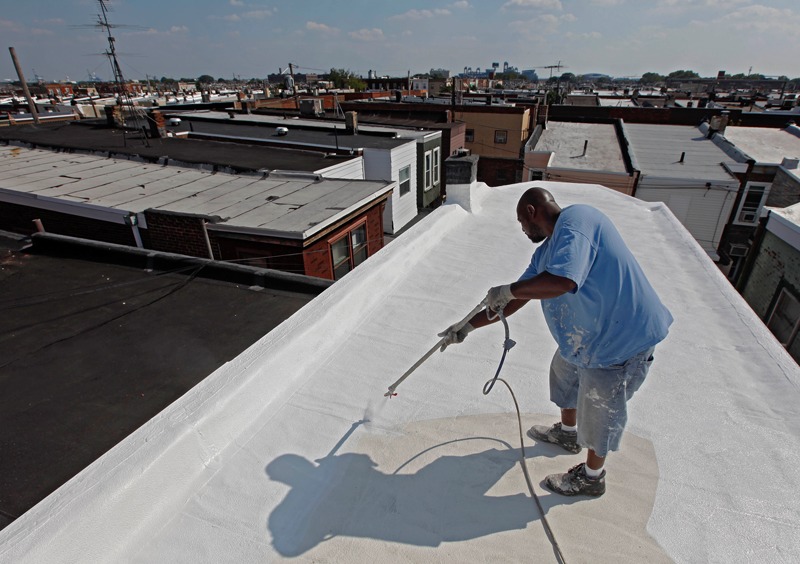
A worker applies a coating of white paint to the roof of a row home in Philadelphia, 19 Aug 2010. Reflective white roofs have been proposed as one way to reduce reliance on air conditioning, although it’s uncertain whether they are suited for all climates. © AP Photo/Matt Rourke

Implementation of adaptive measures involves a separate level of challenges because policy decisions must consider cost-effectiveness. Yet there has been no thorough economic analysis of temperature-associated health costs and public health adaptation options, and policymakers hesitate to invest scarce financial resources in measures whose benefits are ambiguous. Adding to the uncertainty, the actual severity of future weather cannot be predicted—and a 4°C increase in average temperature will create a situation different from that of a 2°C increase—so precisely quantifying the expected economic strain is difficult. Finally, although adaptation costs money now, benefits may not be realized for years.

Preliminary research has laid some groundwork for determining the value of adapting to a changing climate. For example, the “statistical life” concept assigns a monetary value to a year of life, which enables policymakers to better gauge whether an intervention is cost-effective. Additionally, some research has shown that measures such as heat–health warning systems can save both lives and health care costs.

Public health researchers also recognize that solutions to climate-related problems may also benefit health—for instance, a community designed so that residents can safely walk and bike to shop and work not only reduces greenhouse gas emissions but offers the cardiovascular benefits of physical exercise, which in turn may further protect individuals against extreme heat. Such multibenefit solutions might ultimately prove the most cost-effective, but they need to be better understood and quantified before they can be enacted.

